# Dispersal–diversity feedbacks and their consequences for macroecological patterns

**DOI:** 10.1098/rstb.2023.0131

**Published:** 2024-06-24

**Authors:** Adriana Alzate, Oskar Hagen

**Affiliations:** ^1^ Aquaculture and Fisheries Group, Wageningen University and Research, Wageningen, The Netherlands; ^2^ Naturalis Biodiversity Center, Leiden, The Netherlands; ^3^ German Centre For Integrative Biodiversity Research (iDiv) Halle-Jena-Leipzig, Leipzig, Germany; ^4^ Department of Ecological Modelling, Helmholtz Centre for Environmental Research GmbH – UFZ, Leipzig, Germany

**Keywords:** dispersal, diversity, macroecology, macroevolution, species interactions

## Abstract

Dispersal is a key process in ecology and evolution. While the effects of dispersal on diversity are broadly acknowledged, our understanding of the influence of diversity on dispersal remains limited. This arises from the dynamic, context-dependent, nonlinear and ubiquitous nature of dispersal. Diversity outcomes, such as competition, mutualism, parasitism and trophic interactions can feed back on dispersal, thereby influencing biodiversity patterns at several spatio-temporal scales. Here, we shed light on the dispersal–diversity causal links by discussing how dispersal–diversity ecological and evolutionary feedbacks can impact macroecological patterns. We highlight the importance of dispersal–diversity feedbacks for advancing our understanding of macro-eco-evolutionary patterns and their challenges, such as establishing a unified framework for dispersal terminology and methodologies across various disciplines and scales.

This article is part of the theme issue ‘Diversity-dependence of dispersal: interspecific interactions determine spatial dynamics’.

## 1. Introduction

Dispersal is a fundamental process modulating all aspects of diversity, including taxonomical, functional and phylogenetic diversity, at several spatial (alpha, beta and gamma diversity) and temporal scales [1–6]. At short time scales, dispersal mediates colonization and adaptation to new or changing environments [7,8], whereas, at long time scales, dispersal shapes large-scale biodiversity patterns (such as species geographical distributions or latitudinal diversity gradients) [9–12], through speciation and extinction [13–15]. At the local scale, dispersal plays an essential role in community assembly and can shape alpha-diversity patterns by facilitating colonization and preventing local extinctions [7,16–18]. At larger spatial scales, variation in dispersal can lead to changes in overall beta and gamma diversity [19–21].

**Figure 1.**
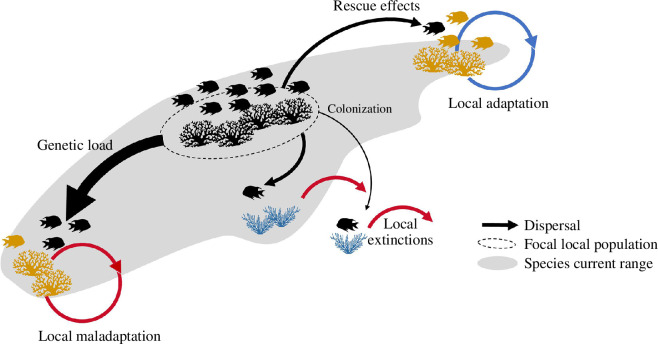
Dispersal affects colonization–extinction dynamics at a short time scale. We depict the effects of dispersal for a species of fish along its current range (grey). Black arrows denote the direction of dispersal from a focal local population (delimited by a dashed line) to other populations at the range borders (yellow and blue coral) as well as to previously unoccupied and unsuitable sites (blue coral) outside as well as within its range. Arrow thickness denotes the strength of dispersal. Red and blue arrows denote negative and positive effects, respectively. Arrows in circles indicate that the impact of dispersal happens through time. Low levels of dispersal often lead to smaller populations characterized by reduced genetic diversity and increased extinction risk. Intermediate levels of dispersal provide demographic and genetic rescue, allowing populations to colonize suboptimal habitats, such as at the range borders, by promoting local adaptation (yellow fish on yellow coral, top right). Conversely, high levels of dispersal might lead to maladaptation owing to genetic load (black fish on yellow coral, bottom left). However, note that dispersal is assumed to occur randomly in this scenario. When dispersal is context- or condition-dependent, it can lead to habitat matching and promote local adaptation even with high dispersal rates [22]. Figure adapted from Alzate [23]. Coral icons created by Agne Alesiute from Noun Project.

The effects of dispersal on colonization, extinction, adaptation and speciation can be positive or negative, depending on its strength and whether dispersal is constant or intermittent. Dispersal can have a positive affect on colonization and adaptation by providing demographic and genetic rescue that buffers demographic stochasticity and prevents extinction (figure 1) [8]. In suboptimal environments, populations can endure through source–sink dynamics [24–28], but high dispersal levels might keep populations maladapted to the local conditions, e.g. by genetic load (figure 1) [8,28–31], negatively affecting adaptation and range expansion, while preventing speciation at the macro scale [32–34]. Nevertheless, in the case of non-random dispersal (condition- and context-dependent dispersal), higher dispersal rates might increase local adaptation [22,35], reinforcing rather than counteracting evolutionary differentiation. Despite all possible directions that dispersal can affect diversity, dispersal-assembled communities have been reported to attain higher species richness than those assembled by niche-based processes alone, suggesting that many more species can coexist transiently (unstable coexistence), reliant on continuous migration or mass effects [36–39].

While the impact of dispersal on diversity is widely recognized, our understanding of the extent to which diversity, in turn, influences dispersal remains limited. The relationship between diversity and dispersal can be described as a feedback loop since their interaction can affect both ends simultaneously [40]. An increase in species diversity in a community likely leads to a rise in the number and variety of species interactions, resulting in complex positive and negative feedbacks, which in turn, might significantly affect dispersal patterns and their evolutionary trajectories [39,41–43]. Biological interactions can exert selective pressure on various species traits, which can ultimately influence dispersal strategies and evolution [44–48]. The intensity and nature of these interactions, e.g. facilitation (positive), predation, competition and parasitism (negative) are expected to shape biodiversity through dispersal-mediated feedback loops. Feedback loops are recognized as a source for a variety of biodiversity patterns [40], while dispersal, in particular, has been shown to be central for eco-evolutionary feedbacks across hierarchical levels and scales [42]. Thus, although dispersal–diversity feedbacks are crucial for biodiversity patterns, their consequences, particularly at larger spatial and temporal scales, remain to be elucidated.

Here, we describe feedbacks between dispersal and (taxonomic) diversity and examine how these feedbacks might impact macroecological patterns. First, we highlight the challenges of studying dispersal because of its dynamic, context-dependent and ubiquitous nature as well as the differences in scales that dispersal operates. Second, we provide an overview of possible diversity–dispersal feedback loops. Third, we provide an overview of the consequences of diversity–dispersal feedback loops for macroecological patterns, challenges of studying them and some potential relevant questions in the field.

## 2. The dynamic-, ubiquitous- and context-dependent nature of dispersal

Dispersal, ‘any movement of individuals or propagules with potential consequences for gene flow across space’ [49], is a dynamic process, encompassing three stages (i.e. departure, transfer and settlement; defined in the glossary in table 1) [49,51]. Ultimately, dispersal is the combined effect of different dispersal-related traits, or a dispersal syndrome [52,53] acting at different life stages within a biotic and abiotic spatiotemporal context. Dispersal outcomes are dependent on a multitude of factors, thus understanding the complexity of the dispersal process, including its drivers and consequences, is essential for a thorough comprehension of ecological and evolutionary dynamics. For instance, selecting ecologically relevant dispersal proxies is crucial, as it significantly impacts the outcomes and interpretation of scientific research [19]. This effect of the choice of dispersal proxy has been shown when studying the determinants of species' geographical distributions, by showing that the effect of dispersal on species range heavily relies on the particular dispersal proxy employed, even among species within the same taxonomic group [19]. The influence of choosing relevant traits extends beyond research explicitly focused on dispersal, affecting a broad spectrum of ecological studies [54,55]. A meticulous selection of dispersal proxy plus the understanding of the nuances of dispersal processes within various ecological and evolutionary contexts can improve and inform models. For example, having dispersal kernels dependent on local diversity properties (i.e. quantity and quality of biotic interactions) can lead to a better understanding of which traits and syndromes are relevant in specific contexts (e.g. group of organism, spatial and temporal context) [56,57].

**Table 1.** Glossary of main terms used in this study.

term	definition
feedback loop	a process in which a system's outputs (updated state) are routed back and used as inputs. Feedback loops can be positive or negative depending on how they change the input. Positive feedback loops tend to enhance or amplify changes, while negative feedback loops tend to dampen or buffer changes
departure stage	initial phase of dispersal, The decision to leave or leaving the natal site. Usually considered a departure probability, which might be triggered by kin competition, inbreeding avoidance, density dependence or habitat heterogeneity
transfer stage	movement from the location of emergence (natal site/site of emergence) to a new location (site of reproduction)
settlement stage	successful arrival at a new site
colonization	establishment in an unoccupied new site for multiple reproductive cycles. Successful colonization depends not only on the successful sequence of all the dispersal stages (i.e. departure, transfer and settlement) but also on ecological suitability (i.e. biotic and abiotic niche)
dispersal-related traits	characteristics or attributes of an organism (i.e. that can be influenced by the environment and shaped by evolutionary history [50]) with a potentially positive effect on dispersal (in one or more stages of dispersal). These traits can be morphological, physiological, life-history or behavioural and are linked to movement (e.g. body size, propagule size, flight ability), endurance (tolerance to conditions, energetic reserves) or colonization success (fast generation times, asexual reproduction) of individuals

Because dispersal influences biodiversity across various spatial and temporal scales, the scale of analysis is a key factor when understanding the impacts of dispersal on diversity [2,58–60]. For instance, the effect of dispersal on alpha, beta and gamma diversity has been shown to be scale-dependent [61,62] and the scale at which dispersal takes place (local, intermediate or global) can result in different species richness–spatial scale relationships [2]. At local spatial scales, dispersal can initially enhance diversity before its effect plateaus, while at broader spatial scales, such as metacommunities or landscapes, dispersal tends to negatively affect diversity [39,43]. The interaction between dispersal, niche overlap and selective predation can sustain levels of species richness, which otherwise would be limited to individuals moving from high- to low-density areas, also referred to as mass effects [62]. Moreover, theoretical work by Mouquet & Loreau [20] suggests a hump-shaped relationship between dispersal and alpha diversity while beta and gamma diversity decline as dispersal increases. Haegemen & Loreau [63] further elaborate on this theory, demonstrating that the dispersal–diversity relationship can also increase monotonically, depending on the combined effects of consumer and resource dispersal on a metacommunity model. Additionally, Grainger & Gilbert [64] conducted a meta-analysis of experimental results, indicating that dispersal–diversity relationships may vary depending on methodological aspects. Furthermore, studies emphasize the need to consider various contextual and methodological factors when investigating dispersal–diversity relationships [13,17,43,58,61,62,65,66]. De Bie *et al*. [65] suggest that the conclusions drawn from dispersal studies may be contingent on the spatial and temporal scales at which the research is conducted, stressing the importance of considering scales when evaluating the effects of dispersal on diversity patterns. These findings illustrate that the effects of dispersal on biodiversity are scale-dependent, highlighting the complexity of dispersal mechanisms in shaping diversity patterns in ecological landscapes.

Dispersal is central to various ecological and evolutionary theories, such as the Island Biogeography Theory [67], the neutral theory of biodiversity and biogeography [68], metapopulation theory [69] and metacommunity theory [21]. Despite its key role in ecology and evolution, particularly in the context of eco-evolutionary feedback loops [42], studying dispersal presents significant challenges. These challenges arise from its dynamic nature, dependence on context and nonlinear characteristics; *sensu* Robertson [67]. The widespread recognition of dispersal has led to numerous similar definitions [49,51,70–72]. Consequently, the interpretation, measurement and study of dispersal vary significantly across and within different scientific disciplines [73,74]. These differences are likely given partially by the fact that dispersal is equally important in explaining both short and large temporal variations in biodiversity. For instance, the maintenance of locally less adapted populations can be attributed to continuous immigration (demographic rescue), whereas allopatric speciation is accounted for by extended periods of halted gene flow. Consequently, subtle differences in the conceptualization of dispersal, but more importantly the highly conceptual or abstract definitions are difficult to avoid. This results in various methodological approaches and the use of dispersal proxies, making comparisons within and between theoretical and empirical work more challenging.

From a theoretical macro perspective, simulating dispersal is challenging because theoretical models need to capture the complex dynamics of organism movement and interaction across diverse landscapes and evolutionary timescales. When constructing theoretical models, researchers often use probability functions, known as dispersal kernels or diffusion principles, to simulate the movement and mixing of organisms for random dispersal [75]. The detail in representing dispersal processes tends to decrease when increasing the spatial or temporal resolution of theoretical models: from individual-based to population-based, from metacommunity to macroevolutionary and from spatial implicit to explicit. These distinctions often intersect; for instance, metacommunity models typically focus on individuals and cover narrower temporal and spatial scales than macroecological or macroevolutionary models. The latter often deal with populations over extensive periods of time and vast areas, sometimes accounting for phenomena like continental drift. Consequently, models reconstructing macroevolutionary processes and patterns need to integrate significant environmental transformations, often simplifying many complex, cumulative and nonlinear processes related to dispersal. Simplification is necessary but problematic, as it must approximate the outcomes of rare, complex and highly stochastic events that have occurred over thousands of years. Such events are related to, but not limited to, variations in fecundity, propagule pressure and colonization success [1,74,76]. These challenges emphasize the inherent difficulties in creating models that accurately reflect macroevolutionary patterns, necessitating abstractions that can sometimes obscure the detailed dispersal processes, especially in the light of eco-evolutionary feedbacks.

Empirical assessments of the causes and consequences of dispersal mirror theoretical studies into comparability issues. Experimental approaches often manipulate variables such as the number or frequency of dispersing individuals [29,77], control immigration by implementing dispersal barriers [39] or alter the distance between target and source habitats [78]. Empirical studies, in general, rely on proxies, such as dispersal-related traits or dispersal syndromes, to investigate the effect of dispersal on ecological and evolutionary patterns [19,73]. Nonetheless, quantitative measures of dispersal exist and involve employing techniques like mark–recapture, radio tracking or genetic analysis that can be used to estimate distances between parents and offspring [79–81]. In studies of comparative macroecology or macroevolution, dispersal is usually assessed at the species level. This is often summarized using continuous dispersal-related traits (e.g. average or maximum body size, seed count, wing measurements) or categorical trait states (e.g. wind versus animal dispersal in plants or pelagic versus non-pelagic eggs in reef fishes) [19,33,82,83]. The various methodologies and measurements used in these studies reveal the challenges in understanding the contributions of dispersal to biodiversity. Factors such as inter-individual variability and dispersal plasticity further complicates our understanding of the topic.

Dispersal exhibits significant inter-individual variability, a fact which is widely recognized by the scientific community today [35,71,84]. The dispersal behaviour of organisms is influenced by their unique phenotypes and the specific environments they encounter. This results in dispersal patterns that are closely linked to the context and/or phenotype, influencing each of the three dispersal stages (defined in the glossary in table 1). The effect on these stages can vary significantly depending on both the external environmental pressures and the internal conditions of the dispersing individuals. This shows the complex relationship between genetics, phenotype and environment in shaping dispersal strategies. Additionally, dispersal can be plastic, varying significantly based on environmental conditions and biotic contexts [45,48,85,86]. Increased dispersal rates may result from encountering new environments at population edges, as is the case for the invasive cane toads (*Rinella marina*), for which toads translocated from the range core to the invasion front exhibit enhanced dispersal [86]. This increase in dispersal is suggested to result from lacking information on the distribution of critical resources, such as refuge, water, food or predation risk [86]. Spatial sorting, a process where faster-dispersing individuals aggregate at the leading edges of expanding populations, can also further impact traits within these frontiers. While we acknowledge the role of the environment in the evolution and adaptation of organisms [87], for the purposes of this discussion, we do not examine the feedback loops between organisms and their physical surroundings.

## 3. Diversity–dispersal feedback loops

**Figure 2.**
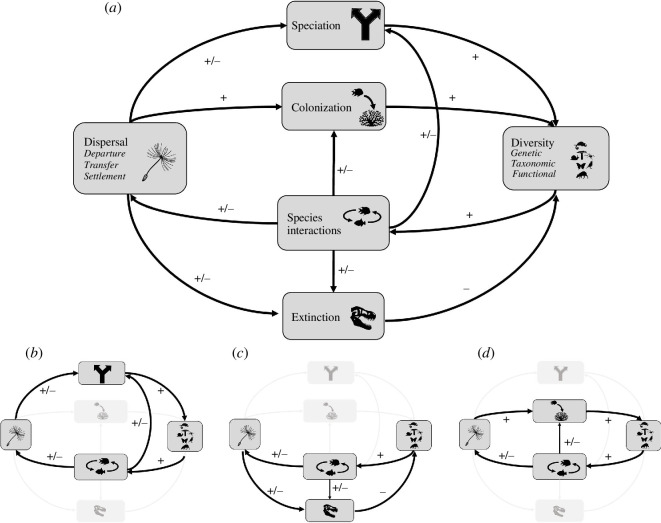
Possible feedbacks between dispersal and diversity (*a*). Black arrows denote the multiple pathways in which dispersal can affect diversity. We highlight three feedback loops via dispersal direct effects on speciation (*b*), extinction (*c*) and colonization (*d*) In turn, diversity indirectly affects dispersal by changing the quantity and the type of species interaction. Arrows denote the pathway direction and signs denote whether the effects are positive (+), negative (−) or both (+/−). Coral, fossil and dandelion icons created by Agne Alesiute, Raf Verbraeken and Olena Panasovska from Noun Project.

Dispersal can drive and respond to ecological and evolutionary processes, shaping biodiversity dynamics at its core. Dispersal influences species diversity via its effects on colonization, extinction, adaptation, speciation, priority effects and species interactions [7,8,16,17,88,89] (figures 1 and 2). In turn, diversity influences dispersal through quantitative and qualitative changes in biological interactions that exert selection on dispersal-related traits (or syndromes), potentially influencing the dispersal process and dispersal evolution [44–48,90–92]. Such back-and-forth effects between dispersal and diversity are examples of feedback loops (see glossary for definition in table 1). Dispersal–diversity feedbacks likely play a crucial role in shaping biodiversity patterns and processes through multiple pathways. Here, we focus on four processes: colonization, speciation, extinction, and species interactions, which are fundamental components responsible for the feedbacks between dispersal and diversity (figure 2*a*). We illustrate three possible loops that highlight the effects of dispersal on diversity via speciation (figure 2*b*), extinction (figure 2*c*) and colonization (figure 2*d*), as well as the effects of diversity on dispersal through its effects on species interactions.

Dispersal can promote speciation (loop figure 2*b*), as it affects local adaptation and ecological specialization by maintaining populations on newly colonized areas and providing demographic and genetic rescue (figure 1). Similarly, diversity affects speciation, showing negative feedbacks between lineage diversity and diversification [93]. High levels of dispersal can impede speciation by maintaining gene flow between populations, hindering local adaptation and genetic divergence (figure 1). However, discussions about how high dispersal levels can hinder local adaptation often focus on the idea that dispersal is random in relation to the environment and the phenotypic and genotypic characteristics of individuals. Nevertheless, non-random dispersal, also known as context- and condition-dependent dispersal, which is seen in many species, might lead to increased dispersal rates that support local adaptation [22,35]. While speciation is the ultimate driver of species diversity [18], its effect on species interactions might take an extendend timeframe to become apparent. In contrast, the influence of colonization events—where species originate from a different location—on species interactions may be more noticeable because those events are easier to observe than speciation. This difference is mainly owing to the different timeframes over which colonization and speciation operate, making difficult the detection of speciation’s impact on species interactions.

Dispersal indirectly influences diversity via its effect on extinction (loop figure 2*c*). Dispersal can maintain diversity by decreasing extinction risk via source–sink dynamics [24–28] and mass effects [94,95]. However, dispersal can decrease diversity by introducing new species into established communities that potentially leads to local extinctions or hinders speciation [34] (but sometimes triggers speciation [96]). This effect may occur as a result of competitive exclusion, predation or other forms of negative interspecific interactions, where the incoming species outcompete, prey upon or otherwise negatively impact existing species. Additionally, local extinction might be triggered by changes in the environment, e.g. as caused by the arrival of ecosystem-engineer species [87]. Changes in biodiversity owing to extinction events can significantly alter dispersal dynamics within ecosystems. For example, when a top predator is removed, resulting in prey overpopulation and resource depletion, surviving species may be forced to disperse to new areas in search of food, altering their traditional movement patterns [97–100]. Similarly, the loss of key pollinators could disrupt food webs, prompting species that relied on those pollinated plants for food to expand their search for resources, thus modifying their dispersal routes [101,102]. These shifts in dispersal are critical for understanding ecosystem resilience and the cascading effects of biodiversity loss. Furthermore, many of these interactions are nonlinear. For example, the final proportion of dispersed seeds is highest in areas with intermediate use by scatter-hoarders [103]. Consequently, the quantity and quality of biotic interactions, considering scale-dependent changes in landscape pattern, species dispersal and establishment characteristics, are relevant to many issues in community ecology, invasion biology and conservation biology.

Successful colonization (loop figure 2*d*) can lead to range expansions with possible consequences for all levels of diversity (figure 2). Taxonomic, functional and genetic diversity might increase if we assume coexistence or additional environmental variability leading to higher genetic/functional variability [104,105]. In the case of an increase in diversity, diversity outcomes, such as competition, predation and mutualism, can thus directly impact dispersal-related traits and ultimately dispersal outcomes and diversity patterns [106,107]. There are several examples of these feedbacks in nature. For instance, high dispersal can evolve under competition in insects, birds and mammals [108,109]. Allometric, genetic and developmental constraints might lead to traits associated with potential negative effects of competition on dispersal [70,110,111]. Parasitism can increase dispersal by triggering dispersal motivation (departure stage of dispersal) or reduce dispersal by negatively affecting body condition and shape in insects, which results in reduced dispersal ability and probability [44,90]. Frugivory can exert selection on dispersal-related traits of plants [112]. For instance, the extinction of megafauna frugivores (mutualism) has resulted in reduced dispersal and recruitment as is the case for palms of the Old World which evolved smaller fruits [82]. This has consequences for future range expansion, maintenance of their current range and, ultimately, diversity patterns by modifying the species’ dispersal potentials [106,107]. Symbiotic relationships such as the ones between plants and mycorrhizal fungi can influence plant dispersal [92]. For instance, orchids and some pteridophytes produce tiny, wind-dispersed seeds or spores that depend heavily on fungi for early growth. Thus the presence (or co-dispersal) of symbiotic mycorrhizal fungi is crucial for establishment (settlement stage of dispersal).

## 4. Consequences for macroecological patterns

Macroecology, which is the study of large-scale patterns and processes, traditionally relies on broad-scale observations and generalizations [113,114]. Dispersal often plays a central role in explaining several macroecological patterns, such as species abundance distributions [115], species–area relationships [116], latitudinal diversity gradients, species geographical distributions [77,117–119] and islands’ biodiversity patterns [67,120]. Dispersal–diversity feedbacks likely influence these patterns and overlooking them could profoundly impact our understanding of global diversity patterns. Recognizing the effects of these feedbacks also requires integrating micro- and macroecological, as well as evolutionary, mechanisms and processes [121]. Harmonizing theories and incorporating data from various disciplines, a more comprehensive array of taxa, locations and interaction types is a way forward for more interdisciplinary macroecological research [122].

Interspecific interactions, such as antagonism and mutualism across trophic levels, play an important role in shaping large-scale richness patterns and ecological network structures [123]. For instance, Pigot and Tobias [124] showed that the distribution of birds is highly influenced by biotic interactions, particularly competition, across various spatial and temporal scales. Other studies have shown that the absence of mutualistic relationships, such as those between plant and animal pollinators or plant and arbuscular mycorrhizal fungi, on oceanic islands, explains variation in plant richness better than traditional factors like island size or isolation, as proposed by the Island Biogeography Theory [125]. Additionally, this mutualism filter (a decline in species richness owing to the absence of mutualist partners) has a more pronounced effect in the tropics, resulting in a weaker latitudinal diversity gradient. This demonstrate the importance of species interactions in explaining large-scale diversity patterns [125]. Parasitism can also influence food-web structure, trophic relationships and dispersal [44,45,126]. Spatial variation in species composition and resulting species interactions can affect population fitness with possible consequences to dispersal processes. Furthermore, geographically variable biotic interactions can have important implications for species ranges [127], with effects on macroevolutionary outcomes, such as trait evolution and diversification [128], which can feedback on dispersal, diversity patterns and phylogenies [129].

Understanding and predicting the impacts of the diversity–dispersal feedbacks on large-scale biodiversity patterns pose various challenges, many of which are associated with the scaling of spatiotemporal processes. For example, multiple assumptions about dispersal mechanisms must be made when modelling, particularly in the presence of dynamic landscapes and species interactions. While large-scale patterns are crucial for understanding dispersal–diversity feedbacks in macroecology, it is crucial to recognize the value of investigating smaller-scale processes such as accounting for or considering upscaling microevolutionary dynamics into macroevolutionary predictions [130]. Bridging this scale gap would involve using consistent dispersal vocabulary within disciplines and can help us understand how intrinsic properties of organisms and geographical settings affect population-level and microevolutionary processes that ultimately change macroevolutionary patterns [130,131]. Moreover, while uncertainties on environmental and climatic reconstructions still pose challenges, predicting the behaviour of feedback loops is crucial for improving macroevolutionary models. These models can then be used to further identify underlying processes and improve our understanding of the relationship between dispersal–diversity ecological and evolutionary components of feedback loops. Although there are many more mechanisms impacting dispersal–diversity feedbacks than the ones presented here, further studies are needed to advance our understanding of how dispersal–diversity feedbacks operate (for some questions, see box 1).

Box 1. **We present 10 questions to investigate feedbacks between dispersal and diversity, as well as the patterns and consequences of these interactions.**

1. *How do dispersal-related traits adapt in response to shifts in biodiversity, and how do these adaptations feedback into biodiversity dynamics, influenced by macro-environmental gradients such as latitudinal variations*? This question explores the link between dispersal traits, biodiversity and macro-environmental patterns.
2. *Are there discernible signatures of species interactions within gradients of species richness*? This question could explore whether there is a correlation between dispersal and local–regional species richness, and whether this is driven by a direct influence of diversity (species interactions).
3. *How does the coevolution of plants and animal dispersers alter macroecological patterns*? This question can be explored by building theoretical models that include feedbacks between dispersal and diversity. Models predictions can be compared with empirical data, and by using empirical comparative analyses across regions with varying coevolutionary dynamics and feedbacks.
4. *How can the deliberate choice of dispersal-related traits or dispersal syndromes fundamentally change conclusions and expectations on large-scale biodiversity patterns*? This question explores how the use of different dispersal proxies (or syndromes) can impact the outcomes of the macroecological patterns. Also whether these differences are consistent across patterns or are trait- and pattern-dependent.
5. *Do dispersal–diversity feedbacks behave similarly when comparing different aspects of diversity*? This question can explore whether the different feedbacks and pathways are consistent when using taxonomic, functional or phylogenetic diversity.
6. *How do feedbacks behave depending on the type of biotic interaction, and how can even the same type of interaction produce different feedbacks*? Do we observe the same effects of biotic interactions (e.g. facilitation, frugivory, competition) on dispersal when we look at local, regional or global scales?
7. *Can we forecast or hindcast dispersal evolution under different invasion ecology scenarios*? How does increased competition resulting from invasive species impact local ecological dynamics and alter dispersal patterns? Are there general trends in dispersal-related trait evolution in invasion histories?
8. *Integrative models of dispersal and diversity across spatial and temporal scales*. How can we develop and validate integrative models that accurately represent dispersal–diversity feedback loops across multiple spatial and temporal scales? This research direction would aim to bridge microevolutionary with macroevolutionary dynamics, addressing the challenges of scaling.
9. *Dispersal-driven changes in interaction networks*. How does the alteration of dispersal patterns, driven by changes in species diversity and densities, affect the structure and function of species interaction networks over time? This aims at investigating the cascading effects of altered dispersal on mutualism, competition, predation and other interactions while considering shifts in interaction signs according to mass effects (e.g. when mutualisms become antagonists when densities are high).
10. *Feedback loops in species range shifts*. In the context of climate change and habitat fragmentation, how do feedback loops between dispersal and diversity manifest in species range shifts? This question explores the role of dispersal–diversity feedbacks in facilitating or hindering species' ability to track suitable habitats, as influenced by rapid environmental changes.

Modelling and demonstrating the feedback mechanisms poses a significant challenge. While we have illustrated how individual components interact with each other based on theoretical and empirical evidence, understanding the system as a whole remains a challenge when all these pathways are integrated. Although incorporating these concepts into theoretical models and generating predictions for macroecological patterns might be relatively straightforward, finding generalities that can also be used for predictions, hindcasts and modelling of conservation scenarios is a more difficult challenge [132,133]. Furthermore, empirically verifying that these patterns directly result from these feedback mechanisms is even more challenging, as establishing causality can be difficult.

## 5. Conclusion

We explore the relationship between dispersal and diversity and how their feedbacks might influence macroecological patterns. Dispersal, a fundamental process in shaping biodiversity and central to most ecological/evolutionary disciplines, operates through multiple pathways and scales, influencing colonization, adaptation, speciation and species interactions. The concept of dispersal–diversity feedbacks is critical to understand how these two ecological forces interact, generate and maintain macroecological patterns. Investigating the complexity of diversity–dispersal feedbacks poses various challenges associated with scaling spatiotemporal processes and using a common language. We emphasize the importance of reaching a consensus on the measurement and metrics of dispersal across various research fields such as improving the simplifications of many of the complex, cumulative and nonlinear processes related to dispersal. Ignoring these feedback loops can obscure the causes of macroecological patterns, as dispersal evolution and the emergence or the disruption of interspecific interactions can have potential consequences for global diversity patterns. Incorporating microevolutionary dynamics into macroevolutionary models and adopting spatially explicit models continue to be small but necessary steps towards addressing these challenges and gaining a more accurate understanding of how dispersal shapes biodiversity and how biodiversity shapes dispersal.

## Data Availability

This article has no additional data.
